# Traditional ecological knowledge-based assessment of threatened woody species and their potential substitutes in the Atakora mountain chain, a threatened hotspot of biodiversity in Northwestern Benin, West Africa

**DOI:** 10.1186/s13002-018-0219-6

**Published:** 2018-03-20

**Authors:** Pierre Onodje Agbani, Konoutan Médard Kafoutchoni, Kolawolé Valère Salako, Rodrigue Castro Gbedomon, Ahuéfa Mauricel Kégbé, Hahn Karen, Brice Sinsin

**Affiliations:** 1 Laboratoire d’Ecologie Appliquée (LEA), Faculté des Sciences Agronomiques (FSA), Université d’Abomey-Calavi, 01 BP 526 Tri postal Cotonou, Bénin; 2 Laboratoire de Biomathématiques et d’Estimations Forestières (LABEF), Faculté des Sciences Agronomiques (FSA), Université d’Abomey-Calavi, 04 BP 1525 Cotonou, Bénin; 3 Botanical Institute, J. W. Goethe-University Frankfurt, Siesmayerstr.70, 60054 Frankfurt am Main, Germany

**Keywords:** Beta-diversity, Atakora mountain chain, Socio-cultural factors, Forest resources, ANOSIM

## Abstract

**Background:**

Atakora mountains in Benin are a unique but fragile ecosystem, harboring many endemic plant species. The ecosystem is undergoing degradation, and the woody vegetation is dramatically declining due to high anthropogenic actions and recurrent drought. This study aimed to (i) assess the diversity of threatened woody species and (ii) identify their potential substitutes in the three regions of the Atakora mountains namely East Atakora, Central Atakora, and West Atakora.

**Methods:**

The data were collected during expeditions on surveyed localities through semi-structured individual interviews. Free-listing was used to record threatened woody species and which were important and why. Alpha-diversity indices were used to assess diversity of threatened and important threatened woody species. A correspondence analysis was used to determine the reason supporting their importance. Differences in species composition were assessed using analysis of similarities. A number of potential substitutes were compared among species using generalized linear models.

**Results:**

A total of 117 woody species (37 families and 92 genera) were identified. The most prominent families were Fabaceae (19.66%), Combretaceae (12.82%), and Moraceae (10.26%), and the richest genera were Ficus (10 species), Combretum (6), and Terminalia (5). Most threatened species differed across regions (East Atakora, Central Atakora, and West Atakora) and included *Afzelia africana*, *Anogeissus leiocarpa*, *Borassus aethiopum*, *Diospyros mespiliformis*, *Khaya senegalensis*, *Milicia excelsa*, and *Pterocarpus erinaceus*. Most socio-economically important species (*K. senegalensis*, *Parkia biglobosa*, *Vitellaria paradoxa*, and *V. doniana*) were used mainly for food, timber, and fuelwood purposes. Old and adult people, and Dendi and Fulfulde sociolinguistic groups had greater knowledge of threatened woody plant species. High intercultural differentiations in species composition were detected between Bariba-Berba and Bariba-Natimba. Knowledge of substitutes also differed across regions with *P. erinaceus*, *Isoberlinia* spp., and *A. africana* being the most cited substitutes.

**Conclusion:**

Basic data was provided here to inform decision and guide efficient management of woody resources. There was evidence that immediate conservation measures are required for some high economic value woody taxa which were critically threatened. Ex-situ conservation of these species while promoting their integration into agroforestry-based systems were recommended. Besides, community-based management programs and community-led initiatives involving knowledgeable people from different horizons will lead to a long-lasting conservation of these threatened resources.

## Background

Forests represent major intergenerational reservoirs of resources sustaining local economy, enhancing food security, providing non-timber forest products and wood, conserving biodiversity, and offering multiple ecosystem services [1–4]. However, forest covers are dramatically declining in West Africa [5, 6], especially in Benin [7, 8], critically threatening the species they host and compromising ecosystem services they provide [9]. Located in the so-called “Dahomey Gap” which is a low-rainfall dry corridor separating Guinean rainforest blocks [10], the Republic of Benin does not have as much forest zones compared to its neighboring countries such as Nigeria, Ghana, and Ivory Coast. Nevertheless, more than 22% of forest areas and 30% of savannah have been lost in Benin from 1995 to 2006 [8] and according to FAO [11], it was 50,000 ha. year^−1^ of forest cover that has been destroyed in the period from 2000 to 2010. A study on land use and land cover change in Central and Northern Benin revealed that land clearance for agriculture, wood extraction, and demographic growth are major causes of forest depletion [12]. Also, illegal settlements and agricultural encroachment on the protected forests [13] and expansion of illegal timber trade are considered as additional threats to the loss of forest resources. Yet, the most serious cause of the extinction of many woody species in the wild in Benin is undoubtedly the selective logging to which they may be subjected [2, 7, 14]. Atakora mountain chain is a region of great ecological and species diversity in the country [15]. It harbors an outstanding flora including three endemic genera (Vitellaria, Pseudocedrela, and Haematostaphis) to the Sudanian zones, two plant species endemics to Benin (*Cyperus beninensis* (Samain, Reynders & Goetgh) Huygh and *Ipomoea beninensis* Akoègninou, Lisowski & Sinsin), and *Thunbergia atacorensis* Akoègninou & Lisowski, an endangered species endemic to the inselbergs of Benin and Togo [16, 17]. Unfortunately, over-logging, exploitation of granitic rock plates, and agricultural exploitation of the mountain chain lead to the degradation of plant communities and threat the integrity of this ecosystem. Furthermore, the study of plant community dynamics across phytogeographical districts revealed a highly regressive ecosystem in the Atakora chain [12]. Thereof, particular attention should be devoted to this area and conservation efforts should target multiple taxa.

The traditional ecological knowledge (TEK) is a valuable component in the sustainable management of resources and conservation of threatened or rare species and biodiversity, as well as protected areas [18–20]. Indeed, it is well established that the knowledge of local people, developed upon the experiences acquired over generations, can complement scientific ecological knowledge for sustainable management of forest ecosystems [21, 22]. Actually, based on ecological knowledge of local people on the decline or the conservation status of different species, many authors have proposed forest management strategies [23–25] and developed methods for using that knowledge efficiently [26].

As a prerequisite for conservation strategies of the Atakora chain, the major aim of this study was to provide the background for efficient management of the threatened woody species in the Atakora mountain chain region in Benin. Specifically, the study aims to (i) assess the diversity of threatened woody species (TWS) based on locals’ traditional ecological knowledge (TEK), (ii) assess the relationship of TEK with socio-demographic factors of informants (age, gender, and sociolinguistic groups), and (iii) identify their potential substitutes in the area.

## Methods

### Study area

**Fig. 1:**
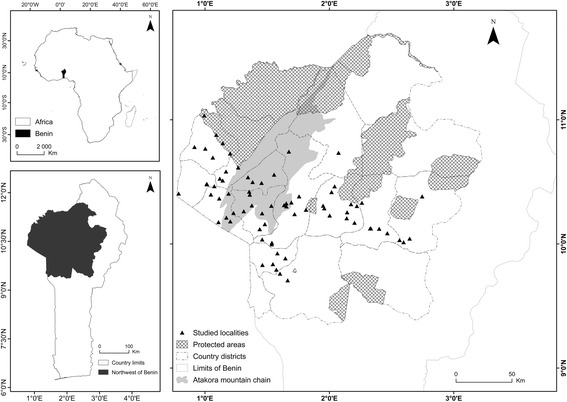
Map showing the study area and indicating the surveyed localities

This study was conducted in 2015, and data presented here were collected over a 6-month period. The study was carried out in the Atakora mountain chain region in Benin (6°–12°50′N and 1°–3°40′E) (Fig. 1). The Atakora chain region includes East Atakora (EA), Central Atakora (CA), and West Atakora (WA) zones. The climate is of Sudanian type and is influenced by the Atakora mountain chain in the state district of Atakora and with a tendency toward a Sahelian climate northward. The rainfall is irregular and unimodal with one rainy season and a dry season which last up to 7 months. The annual rainfall varies between 900 and 1300 mm, and the mean annual temperature is 27 °C [27]. The relief is mountainous with poor sandy, rocky, and encrusted soils and some shallows. Soil is ferruginous. The main sociolinguistic groups encountered in the area are Bariba, Berba, Biali, Dendi, Ditamari, Fulfulde, Lamba, Natimba, Otamari, and Waama [28].

### Sampling and data collection

Twelve state districts belonging to the study regions were selected, and in each district, 2 to 12 localities were randomly selected for the survey (Fig. 1). A total of 267 informants were surveyed throughout the study area, taking into account the geographical location, gender, age, and sociolinguistic group (Table 1). Only informants relatively aged who are expected to have experience and knowledge on the dynamic of woody resources over time were considered. Age of interviewees ranged from 25 to 120 years. The data were collected during expeditions using individual semi-structured interviews and field visits in the selected localities. The questionnaire for the interviews comprised two parts. The first was related to the socio-demographic data of the respondents (name, age, sex, sociolinguistic group, locality) while the second concerned the respondent’s knowledge on the TWS using the free-listing technique. In each locality, interviewees were randomly selected among men and women in different households. However, because of social constraints that made women not very accessible, the study ended up sampling a lot more men than women (16 women and 251 men). Each informant was asked first to list as much threatened woody species s/he knows. In assigning a woody species to as threatened versus not threatened, informants were asked to mainly consider the availability of the woody species through (i) whether they travel more distances or spent more energy to find a particular species that they used to find easily in the past and (ii) whether the extent of the distribution of the woody species has shrunk as compared to its pas extent of distribution. These criteria used for rigorous IUCN assessment [29] are also commonly used to assess species availability in ethnobotanical study (see de Albuquerque [30]). Finally, the informant was asked to mention whether or not each species s/he cited is important and to give the reason of its importance in terms of category of uses. Individual interviews were followed by field visits accompanied with key informants to collect species specimens.

**Table 1:** Sample composition according to location, gender, age, and sociolinguistic groups

		Zone			Total
		EA	CA	WA	
Gender	Women	3	1	12	16
	Men	101	86	64	251
Age	Age < 40	2	15	14	31
	40 ≤ age ≤ 60	41	36	31	108
	Age > 60	61	36	31	128
Sociolinguistic group	Bariba	75	12	0	87
	Berba	0	0	43	43
	Dendi	17	2	0	19
	Fulfulde	12	5	0	17
	Natimba	0	15	15	30
	Otamari	0	23	18	41
	Waama	0	30	0	30
Total		104	87	76	267

*EA* East Atakora, *CA* Central Atakora, *WA* West Atakora

### Data analysis

Collected plant samples were identified at the botanical garden at the University of Abomey-Calavi, Benin, using field herbariums. Data processing consisted in grouping interviewees by sociolinguistic group, gender, age, and zone, then computing descriptive statistics (frequencies, percentages, means ± standard error) for species, genera, and botanical families to draw barplots and generate tables at different levels. Three age groups were created: (a) ≤ 40 years old hereafter called “young,” (b) from 40 to 60 years called “adult” from now on, and (c) ≥ 60 years referred to as “old” from now on. This age categorization followed the United Nations’ World Population Aging 2013 [31] where children and adolescents are under the age of 20 years; young adults (“young” in this study) are between 20 and 39 years of age, middle-aged adults (“adult” in this study) are aged from 40 to 59 years, and older persons (“old” in this study) are aged 60 years or over. To compare the number of threatened, and important species cited by the respondents among age groups, zones and sociolinguistic groups, analysis of variance (ANOVA) or Kruskal-Wallis test was performed when appropriate. ANOVA and the Student Newman Keuls (SNK) post hoc test were used when normality and homoscedasticity assumptions were met, and Kruskal-Wallis test and the Dunn post hoc test when normality and homoscedasticity assumptions were not met [32]. The Dunn test was used as post hoc test instead of the Tukey-Kramer-Nemenyi test because it is appropriate for groups with unequal sizes [33]. Normality and homoscedasticity assumptions were tested using Shapiro-Wilk and Levene’s tests, respectively. The Dunn post hoc test was performed using the package *FSA* [34] in R software [35]. Since the number of women in the study (16) was very unbalanced for making robust inference [36], no statistical comparison was made regarding gender, although descriptive statistics have been reported. To assess the reason supporting the importance of threatening woody species, a correspondence analysis was applied on the contingency table of categories of use and important species. A correspondence analysis was performed using the *FactomineR* package [37]. To determine the most threatened woody species mentioned by the respondents in each zone, the average order of citation was computed for each species and plotted against the frequency of citation of the species. The rationale of using this method relied on the fact that when people are asked to freely list items, they tend to mention the most prominent one first [38, 39]. Most threatened species are species with high frequency of citation and low-average order of citation while least threatened species are species with low frequency of citation and high-average order of citation. Analysis of similarities (ANOSIM) [40] was used to test for differences in threatened and important woody species composition among age group, region, and sociolinguistic group. ANOSIM analysis was performed based on Jaccard dissimilarity distance and 1000 permutations in the package *vegan* [41]. Generalized linear models (GLM) with Poisson (or quasi-Poisson) error distribution were performed to test for differences among regions as regards the average number of substitutes cited by respondents. Relative frequency of citation of substitutes were computed by region and for each of the most threatened woody species in order to determine the most cited substitutes per region and for each TWS. A non-metric multidimensional scaling (NMDS) was used to assess the degree of distinctiveness of the substitute species across the three regions. NMDS was performed in the *vegan* package using the function *metaMDS* and based on Bray distance [42]. Finally, we looked at whether the potential substitutes belong to the same functional group as the substituted species in term of life forms. This was done to assess flexibility in identifying substitutes but also understand whether locals can go over functional group and why.

## Results

### Taxonomic diversity of threatened woody species

**Fig. 2:**
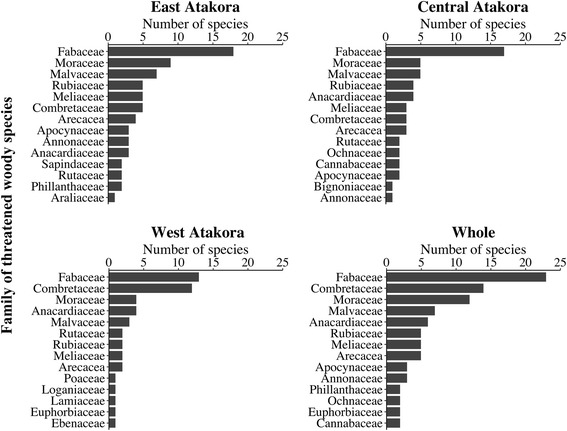
Richer families of threatened woody species in the Atakora mountain region

**Fig. 3:**
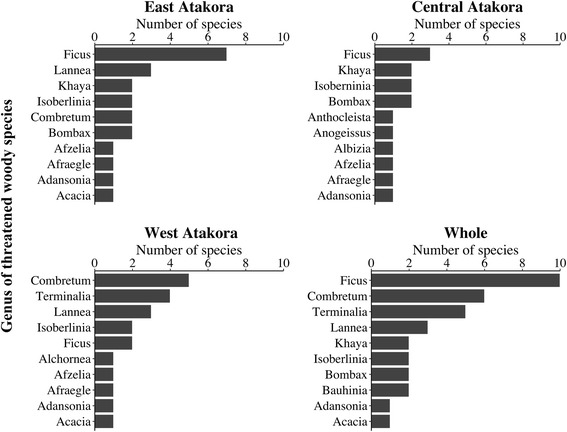
Richer genera of threatened woody species in the Atakora mountain region

A total of 117 species belonging to 92 genera and 37 families were collected and identified as threatened woody species in the study area (Table 2). The most represented family in East Atakora (EA) was Fabaceae with 18 species, followed respectively by Moraceae (9 species), Malvaceae (7 species), Rubiaceae, Meliaceae, and Combretaceae (5 species each) (Fig. 2). In Central Atakora (CA), the richest family was also Fabaceae (17 species), followed by Moraceae (5), and Malvaceae (5). In West Atakora (WA), Fabaceae (13 species) and Combretaceae (12 species) stood respectively first and second as the families with the highest species richness (Fig. 2). Overall, in the study area, the most represented families were Fabaceae (23 species), Combretaceae (15 species), Moraceae (12 species), Malvaceae (7 species), Anacardiaceae (6 species), Rubiaceae (5 species), Meliaceae (5 species), and Arecaceae (5 species), and other families had less than 5 species (Fig. 2). Twenty-six families were represented by only one species (Table 2). The richest genera in EA were respectively Ficus (7), Lannea (3), Khaya (2), Isoberlinia (2), Combretum (2), and Bombax (2). In CA, the most represented genera were respectively Ficus (3), Khaya (2), Isoberlinia (2), and Bombax (2) while in WA, the most represented genera were Combretum (6), Terminalia (4), Lannea (3), Isoberlinia (2), and Ficus (2). Overall, Ficus stood as the first genera with 10 species, followed by Combretum (6 species), Terminalia (5 species), Lannea (3 species), and Khaya, Isoberlinia, Bombax, and Bauhinia each one represented by two species (Fig. 3).

**Table 2:** Threatened woody species collected in the Atakora mountain chain region in Benin

No.	Voucher specimen code	Botanical family	Species	Frequency of citations (%)				CS	
				EA (*n* = 104)	CA (*n* = 87)	WA (*n* = 76)	Whole (*n* = 267)	Benin	IUCN
1	2005	Anacardiaceae	*Haematostaphis barteri* Hook. fil.	0.00	5.75	0.00	1.87	nf	nf
2	2617	Anacardiaceae	*Lannea acida* A. Rich.	8.65	0.00	2.63	4.12	nf	nf
3	1528	Anacardiaceae	*Lannea barteri* (Oliv.) Engl.	1.92	0.00	2.63	1.50	nf	nf
4	1388	Anacardiaceae	*Lannea microcarpa* Engl. & K. Krause	14.42	1.15	35.53	16.10	nf	nf
5	2399	Anacardiaceae	*Sclerocarya birrea* (Sond.) Kokwaro	0.00	5.75	38.16	12.73	nf	nf
6	823	Anacardiaceae	*Spondias mombin* Jacq.	0.00	5.75	0.00	1.87	nf	nf
7	1996	Annonaceae	*Annona senegalensis* Pers.	1.92	0.00	0.00	0.75	nf	nf
8	1749	Annonaceae	*Hexalobus monopetalus* (A. Rich.) Engl. & Diels	8.65	3.45	0.00	4.49	nf	nf
9	372	Annonaceae	*Uvaria chamae* P. Beauv.	0.96	0.00	0.00	0.37	nf	nf
10	1818	Apocynaceae	*Holarrhena floribunda* (G.Gon) T. Durand & Schinz	7.69	0.00	0.00	3.00	nf	nf
11	4640	Apocynaceae	*Saba comorensis* (Bojer) Pichon	4.81	5.75	0.00	3.75	nf	nf
12	3680	Apocynaceae	*Strophanthus hispidus* A.P. De Candolle	9.62	9.20	0.00	6.74	nf	nf
13	344	Araliaceae	*Cussonia arborea* Hochst. Ex A.Rich.	6.73	0.00	0.00	2.62	nf	nf
14	4158	Arecaceae	*Borassus aethiopum* Mart.	83.65	81.61	28.95	67.42	VU	LC
15	4190	Arecaceae	*Elaeis guineensis* Jacq.	41.35	28.74	0.00	25.47	nf	LC
16	3547	Arecaceae	*Hyphaene thebaica* (L.) Mart.	0.00	0.00	17.11	4.87	nf	nf
17	578	Arecaceae	*Phoenix reclinata* Jacq.	13.46	0.00	0.00	5.24	nf	nf
18	4437	Arecaceae	*Raphia sudanica* A.Chev.	36.54	14.94	0.00	19.10	nf	DD
19	3178	Bignoniaceae	*Kigelia africana* (Sprague) Bidgood & Verdc.	47.12	14.94	0.00	23.22	VU	nf
20	4284	Burseraceae	*Commiphora africana* (Rich.) Engl.	2.88	1.15	0.00	1.50	nf	nf
21	4459	Cannabaceae	*Celtis integrifolia* Lam.	4.81	1.15	0.00	2.25	nf	nf
22	940	Cannabaceae	*Chaetachme aristata* Planch.	0.00	5.75	0.00	1.87	nf	nf
23	1531	Chrysobalanaceae	*Maranthes polyandra* (Benth.) Prance	3.85	0.00	0.00	1.50	nf	nf
24	375	Clusiaceae	*Pentadesma butyracea* Sabine	3.85	5.75	0.00	3.37	VU	nf
25	1053	Combretaceae	*Anogeissus leiocarpa* (DC.) Guill. & Perr.	49.04	64.37	92.11	66.29	nf	nf
26	637	Combretaceae	*Combretum adenogonium* Steud. ex A. Rich.	0.00	0.00	15.79	4.49	nf	nf
27	1146	Combretaceae	*Combretum collinum* (Kotschy) Okafor	0.00	0.00	15.79	4.49	nf	nf
28	2583	Combretaceae	*Combretum glutinosum* Perr. Ex DC.	0.00	0.00	15.79	4.49	nf	nf
29	1226	Combretaceae	*Combretum micranthum* G. Don	7.69	4.60	17.11	9.36	nf	nf
30	2456	Combretaceae	*Combretum molle R.* Br. Ex G. Don	0.00	0.00	10.53	3.00	nf	nf
31	1295	Combretaceae	*Combretum platypterum* (Welw.) Hutch. & Dalz.	1.92	0.00	0.00	0.75	nf	nf
32	–	Combretaceae	*Combretum spp*	0.00	0.00	15.79	4.49	nf	nf
33	2560	Combretaceae	*Guiera senegalensis* J.F.Gmel.	0.00	0.00	15.79	4.49	nf	nf
34	701	Combretaceae	*Pteleopsis suberosa* Engl. & Diels	2.88	9.20	2.63	4.87	nf	nf
35	2010	Combretaceae	*Terminalia avicennioides* Guill. & Perr.	7.69	0.00	22.37	9.36	nf	nf
36	1568	Combretaceae	*Terminalia laxiflora* Engl.	0.00	0.00	2.63	0.75	nf	nf
37	1055	Combretaceae	*Terminalia macroptera* Guill. & Perr.	0.00	0.00	2.63	0.75	nf	nf
38	3639	Combretaceae	*Terminalia mollis* M. Laws.	0.00	0.00	2.63	0.75	nf	nf
39	5228	Combretaceae	*Terminalia superba* Engl. & Diels	0.00	0.00	0.00	0.00	VU	nf
40	3127	Dipterocarpaceae	*Monotes kerstingii* Gilg	0.96	0.00	0.00	0.37	nf	nf
41	497	Ebenaceae	*Diospyros mespiliformis* Hochst. Ex A.DC.	50.96	54.02	86.84	62.17	nf	nf
42	2488	Euphorbiaceae	*Alchornea cordifolia* (Shumach. & Thonn.) Müll.Arg.	0.00	0.00	9.21	2.62	nf	nf
43	3138	Euphorbiaceae	*Euphorbia poissonii* Pax	2.88	1.15	0.00	1.50	nf	nf
44	3537	Fabaceae	*Acacia nilotica* (L.) Willd. & Delile	9.62	0.00	13.16	7.49	nf	nf
45	1560	Fabaceae	*Afzelia africana* Pers.	93.27	93.10	42.11	78.65	EN	VU
46	2191	Fabaceae	*Albizia zygia* (DC.) J.F.Macbr.	1.92	4.60	0.00	2.25	nf	nf
47	2091	Fabaceae	*Andira inermis* (Wright) DC.	0.96	0.00	0.00	0.37	nf	nf
48	5163	Fabaceae	*Bauhinia reticulata* DC.	1.92	0.00	0.00	0.75	nf	nf
49	1723	Fabaceae	*Bauhinia thonningii* Schum.	0.00	0.00	6.58	1.87	nf	nf
50	2518	Fabaceae	*Berlinia grandiflora* (Vahl) Hutch. & Dalziel	6.73	9.20	0.00	5.62	nf	nf
51	686	Fabaceae	*Burkea africana* Hook.	9.62	11.49	2.63	8.24	nf	nf
52	2299	Fabaceae	*Cassia sieberiana* DC.	5.77	11.49	6.58	7.87	nf	nf
53	629	Fabaceae	*Daniellia oliveri* (Rolfe) Hutch. & Dalziel	19.23	1.15	0.00	7.87	nf	nf
54	1816	Fabaceae	*Detarium microcarpum* Guill. & Perr.	0.00	0.00	23.68	6.74	nf	nf
55	226	Fabaceae	*Entada africana* Guill. & Perr.	3.85	0.00	0.00	1.50	nf	nf
56	1816	Fabaceae	*Erythrina senegalensis* DC.	1.92	9.20	0.00	3.75	nf	nf
57	2500	Fabaceae	*Faidherbia albida* (Delile) A. Chev.	0.00	1.15	9.21	3.00	nf	nf
58	1277	Fabaceae	*Isoberlinia doka* Craib & Stapf	32.69	11.49	23.68	23.22	nf	nf
59	6038	Fabaceae	*Isoberlinia tomentosa* (Harms) Craib & Stapf	27.88	11.49	23.68	21.35	nf	nf
60	4198	Fabaceae	*Parkia biglobosa* (Jacq.) G. Don	44.23	70.11	65.79	58.80	nf	nf
61	1845	Fabaceae	*Pericopsis laxiflora* (Baker) Meeuwen	12.50	4.60	0.00	6.37	nf	nf
62	1054	Fabaceae	*Prosopis africana* (Guill. & Perr.) Taub.	14.42	19.54	43.42	24.34	nf	nf
63	1690	Fabaceae	*Pterocarpus erinaceus* Poir.	80.77	88.51	85.53	84.64	EN	nf
64	3516	Fabaceae	*Swartzia madagascariensis* Desv.	0.00	3.45	0.00	1.12	nf	nf
65	1715	Fabaceae	*Tamarindus indica* L.	37.50	13.79	26.32	26.59	nf	nf
66	1788	Fabaceae	*Tephrosia vogelii* Hook.f.	0.00	1.15	0.00	0.37	nf	nf
67	1851	Gentianaceae	*Anthocleista djalonensis* A. Chevalier	7.69	9.20	0.00	5.99	nf	nf
68	876	Lamiaceae	*Vitex doniana* Sweet	37.50	47.13	60.53	47.19	nf	nf
69	2053	Loganiaceae	*Strychnos innocua* Delile	0.00	0.00	2.63	0.75	nf	nf
70	2269	Malvaceae	*Adansonia digitata* L.	33.65	17.24	40.79	30.34	nf	nf
71	3984	Malvaceae	*Bombax buonopozense* Beauv.	21.15	18.39	0.00	14.23	nf	nf
72	1765	Malvaceae	*Bombax costatum* Pellegrin & Vuillet	48.08	51.72	51.32	50.19	nf	nf
73	1710	Malvaceae	*Ceiba pentandra* (L.) Gaertn.	62.50	18.39	5.26	31.84	nf	nf
74	4206	Malvaceae	*Cola gigantea* A. Chevalier	7.69	0.00	0.00	3.00	nf	nf
75	1549	Malvaceae	*Sterculia setigera* Del.	7.69	3.45	0.00	4.12	nf	nf
76	2100	Malvaceae	*Triplochiton scleroxylon* K. Schum.	2.88	0.00	0.00	1.12	EN	LC
77	1934	Meliaceae	*Ekebergia capensis* Sparrm.	6.73	0.00	0.00	2.62	nf	nf
78	2136	Meliaceae	*Khaya grandifoliola* C. DC.	19.23	8.05	0.00	10.11	EN	VU
79	2436	Meliaceae	*Khaya senegalensis* (Desv.) A. Juss.	97.12	98.85	98.68	98.13	EN	VU
80	834	Meliaceae	*Pseudocedrela kotschyi* (Schweinf.) Harms	21.15	8.05	2.63	11.61	nf	nf
81	1299	Meliaceae	*Trichilia emetic* Vahl	0.96	0.00	0.00	0.37	nf	nf
82	B163	Moraceae	*Antiaris toxicaria* (Engl.) C. C. Berg	30.77	42.53	46.05	38.95	nf	nf
83	910	Moraceae	*Ficus glumosa* Del.	1.92	0.00	0.00	0.75	nf	nf
84	1275	Moraceae	*Ficus gnaphalocarpa* Steud. ex Miq.	0.00	0.00	26.32	7.49	nf	nf
85	2670	Moraceae	*Ficus ingens* (Miq.) Miq.	0.00	1.15	0.00	0.37	nf	nf
86	1017	Moraceae	*Ficus ovata* D. Don	0.96	0.00	0.00	0.37	nf	nf
87	5183	Moraceae	*Ficus platyphylla* Del.	2.88	20.69	5.26	9.36	nf	nf
88	2430	Moraceae	*Ficus sur* Forssk.	3.85	0.00	0.00	1.50	nf	nf
89	859	Moraceae	*Ficus thonningii* Bl.	0.00	9.20	0.00	3.00	nf	nf
90	994	Moraceae	*Ficus trichopoda* Bak.	1.92	0.00	0.00	0.75	nf	nf
91	1226	Moraceae	*Ficus umbellata* Vahl	4.81	0.00	0.00	1.87	nf	nf
92	2380	Moraceae	*Ficus vallis-choudae* Del.	4.81	0.00	0.00	1.87	nf	nf
93	1476	Moraceae	*Milicia excelsa* (Welw.) C. C.	93.27	72.41	13.16	63.67	EN	VU
94	3350	Myrtaceae	*Syzygium guineense* Keay	17.31	0.00	0.00	6.74	nf	nf
95	518	Ochnaceae	*Lophira lanceolata* Van Tiegh. ex Keay	9.62	11.49	0.00	7.49	nf	nf
96	2666	Ochnaceae	*Ochna schweinfurthiana* F. Hoffm.	0.00	18.39	0.00	5.99	nf	nf
97	1316	Olacaceae	*Olax subscorpioidea* Oliver	2.88	18.39	0.00	7.12	nf	nf
98	4284	Oleaceae	*Chionanthus niloticus* (Oliv.) Stearn	9.62	8.05	0.00	6.37	nf	nf
99	1477	Opiliaceae	*Opilia amentacea* Roxb.	1.92	0.00	0.00	0.75	nf	nf
100	2032	Phillanthaceae	*Uapaca togoensis* Pax	4.81	1.15	0.00	2.25	nf	nf
101	346	Phyllanthaceae	*Margaritaria discoidea* (Baill.) G.L.Webster	1.92	0.00	0.00	0.75	nf	nf
102	2208	Poaceae	*Oxytenanthera abyssinica* (A.Rich.) Munro	15.38	25.29	3.95	15.36	nf	nf
103	196	Polygalaceae	*Securidaca longipedunculata* Fresen.	9.62	4.60	0.00	5.24	nf	nf
104	2240	Proteaceae	*Protea madiensis* (Beard) Chisumpa & Brummit	7.69	0.00	0.00	3.00	nf	nf
105	2065	Rubiaceae	*Breonadia salicina* (Vahl) Hepper & J.R.I.Wood	6.73	2.30	0.00	3.37	nf	nf
106	688	Rubiaceae	*Crossopteryx febrifuga* (Afzel. ex G. Don) Benth.	3.85	0.00	0.00	1.50	nf	nf
107	2541	Rubiaceae	*Gardenia erubescens* Stapf & Hutch.	1.92	1.15	0.00	1.12	nf	nf
108	2089	Rubiaceae	*Mitragyna inermis* (Willd.) Kuntze	1.92	9.20	35.53	13.86	nf	nf
109	2463	Rubiaceae	*Sarcocephalus latifolius* (Sm) E.A.Bruce	6.73	9.20	3.95	6.74	nf	nf
110	1911	Rutaceae	*Afraegle paniculata* (Schum.) Engl.	18.27	54.02	35.53	34.83	EN	nf
111	4500	Rutaceae	*Zanthoxylum zanthoxyloides* (Lam.) B. Zepernick & F.K. Timler	3.85	28.74	19.74	16.48	VU	nf
112	309	Salicaceae	*Oncoba spinosa* Forssk.	0.00	0.00	2.63	0.75	nf	nf
113	872	Sapindaceae	*Blighia sapida* Koenig	14.42	16.09	0.00	10.86	nf	nf
114	261	Sapindaceae	*Zanha golungensis* Hiern	0.96	0.00	0.00	0.37	nf	nf
115	1806	Sapotaceae	*Vitellaria paradoxa* C.F.Gaertn.	49.04	55.17	44.74	49.81	VU	VU
116	1845	Ximeniaceae	*Ximenia americana* L.	13.46	0.00	0.00	5.24	nf	nf
117	2575	Zygophyllaceae	*Balanites aegyptiaca* (L.) Delile	4.81	0.00	18.42	7.12	nf	nf

*EA* East Atakora, *CA* Central Atakora, *WA* West Atakora, *CS* conservation status, *VU* vulnerable, *EN* endangered, *LC* least concern, *DD* data deficiency, *nf* not found

**Fig. 4:**
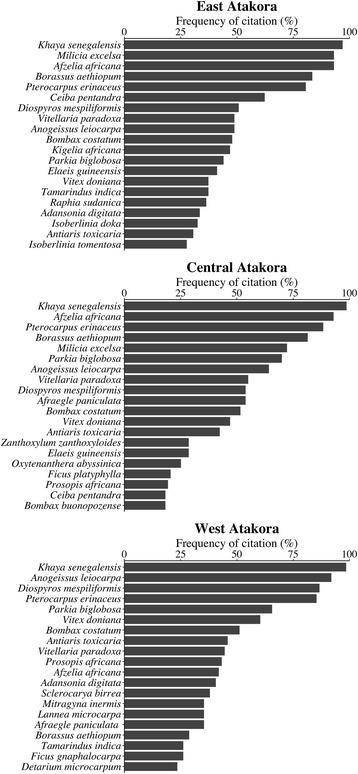
Top 20 more cited threatened woody species

In EA, *Khaya senegalensis* (Meliaceae), *Afzelia africana* (Fabaceae), *Milicia excelsa* (Moraceae), *Borassus aethiopum* (Arecaceae), *Pterocarpus erinaceus* (Fabaceae), *Ceiba pentandra* (Malvaceae), and *Diospyros mespiliformis* (Ebenaceae) were respectively the most cited woody species (cited by at least 50% of informants), while in CA, the most cited threatened woody species were respectively *K. senegalensis*, *A. africana*, *P. erinaceus*, *B. aethiopum*, *M. excelsa*, *Parkia biglobosa* (Fabaceae), *Anogeissus leiocarpa* (Combretaceae), *Vitellaria paradoxa* (Sapotaceae), *Afraegle paniculata* (Rutaceae), *D. mespiliformis* (Ebenaceae), and *Bombax costatum* (Malvaceae). In WA, the threatened woody species most mentioned by respondents were respectively *K. senegalensis*, *A. leiocarpa*, *D. mespiliformis*, *P. erinaceus*, *P. biglobosa*, *Vitex doniana* (Lamiaceae), and *B. costatum*. Three species were commonly more cited in the three regions: *K. senegalensis*, *P. erinaceus*, and *D. mespiliformis* (Fig. 4).

### Most threatened woody species

**Fig. 5:**
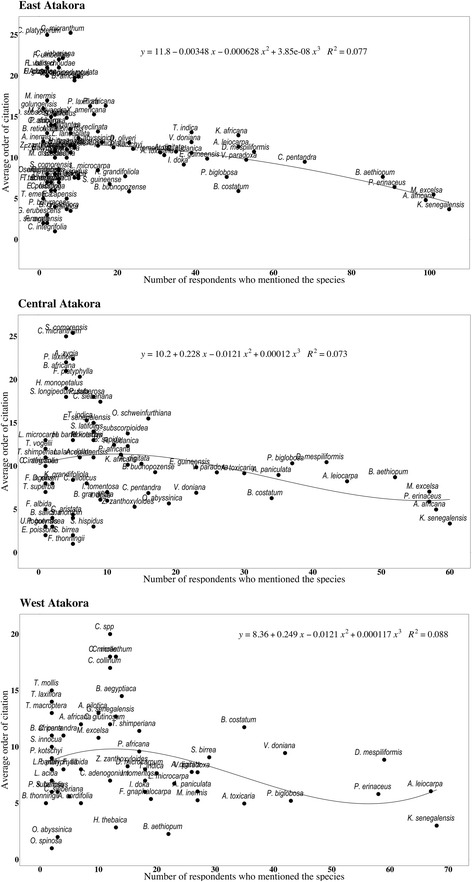
Most threatened woody species in the Atakora chain region of Benin

The most threatened woody species in East and Central Atakora (*K. senegalensis*, *A. africana*, *M. excelsa*, *P. erinaceus*, *and B. aethiopum*) were different from those identified in West Atakora which were *K. senegalensis*, *A. leiocarpa*, *P. erinaceus*, *and D. mespiliformis* (Fig. 5). Therefore, people from East and Central Atakora regions mentioned different woody species as the most threatened compared to people from West Atakora region, except for *K. senegalensis* that was considered as one of the most threatened woody species in all regions.

### Taxonomic diversity of threatened woody species perceived as socio-economically important

**Fig. 6:**
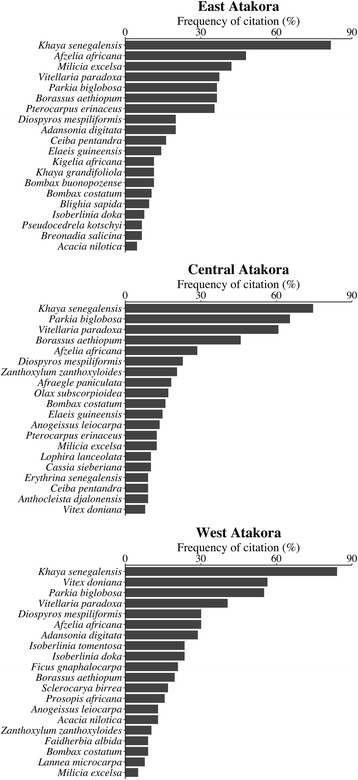
Top 20 threatened woody species more mentioned as important in the Atakora mountain region

Among the inventoried threatened woody species, those that were important for the informants also varied across regions as presented on Fig. 6. For people in East Atakora (EA), *K. senegalensis* was the most important threatened woody species (cited by at least 50% of respondents). The species mentioned as the most important in Central Atakora (CA) were respectively *K. senegalensis*, *P. biglobosa*, and *V. paradoxa*. In West Atakora region (WA), *K. senegalensis*, *V. doniana*, and *P. biglobosa* were the most important. Irrespective of regions, *Khaya senegalensis* was the most important threatened woody species (Fig. 6).

**Fig. 7:**
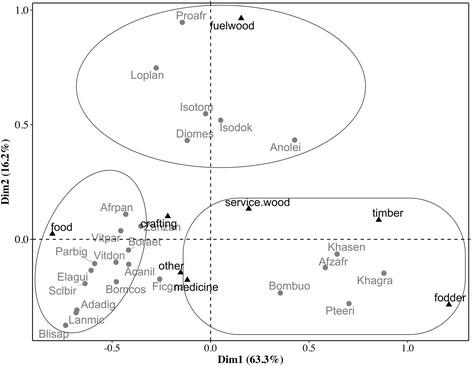
Projection of important threatened woody species in the correspondence analysis system axes formed by use categories

Result from the correspondence analysis performed on important TWS and their use categories indicated that the two first axes encountered for 79.49% of the total variation in the data. The first axis opposed food use category (negative pole) to timber and fodder use categories (positive pole). The second axis was formed by fuelwood use-category in the positive pole (Fig. 7). Projection of the important threatened woody species into the axis system identified three groups of species. The first group included the species used mainly for food which were *Adansonia digitata*, *B. costatum*, *B. aethiopum*, *Blighia sapida*, *Elaeis guineensis*, *P. biglobosa*, *Sclerocarya birrea*, *V. paradoxa*, *V. doniana*, and *Zanthoxylum zanthoxyloides.* The second group was formed by species such as *A. africana*, *Bombax buonopozense*, *K. grandifoliola*, *K. senegalensis*, and *P. erinaceus* not only used mainly for timber and fodder purposes but also as service wood and for medicinal purposes. The third group formed by species mostly used as fuelwood, included *Prosopis africana*, *A. leiocarpa*, *D. mespiliformis*, *I. doka*, *I. tomentosa*, and *Lophira lanceolata* (Fig. 7).

### Threatened and socio-economical important woody species: gender, generation, geographical location, and sociolinguistic group differences

**Fig. 8:**
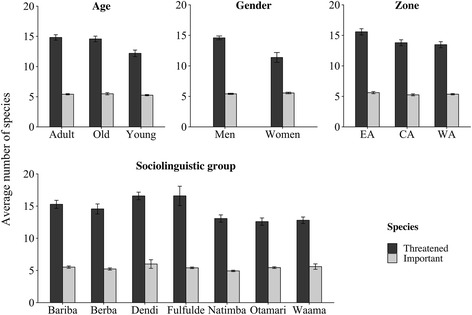
Number of threatened and socio-economically important species mentioned according to socio-demographic factors

The number of threatened woody species (TWS) cited per respondent varied significantly among age categories (ANOVA; *p* = 0.030). Adult (14.82 ± 0.45) and old (14.57 ± 0.47) informants cited more species than younger ones (12.19 ± 0.54; Fig. 8). Men cited 14 ± 0.31 species, and women informant mentioned 11.38 ± 0.81 threatened woody species. The number of species was not compared between genders. Respondents from EA mentioned more threatened species (15.58 ± 0.51) compared to those from CA and WA (13.79 ± 0.49 and 13.47 ± 0.5, respectively). The number of TWS cited per respondent varied also among the sociolinguistic groups (Kruskal-Wallis test; *p* = 0.003). Dendi (16.58 ± 0.59) and Fulfulde (16.59 ± 1.5) people cited higher number of species while Natimba (13.07 ± 0.57), Otamari (12.59 ± 0.57), and Waama (12.8 ± 0.51) cited less species. Bariba (15.28 ± 0.62) and Berba (14.56 ± 0.78) people cited average number of species (Fig. 8).

The number of TWS rated as socio-economically important was not influenced neither by age (Kruskal-Wallis test; *p* = 0.798) nor by region (Kruskal-Wallis test; *p* > 0.05). Women mentioned 5.56 ± 0.13 species as important while men mentioned 5.42 ± 0.1 species. The number of TWS important to people also varied among sociolinguistic groups (Kruskal-Wallis test; *p* = 0.006). Bariba (5.52 ± 0.18), Berba (5.23 ± 0.17), Dendi (6 ± 0.67), Waama (5.6 ± 0.42), and Fulfulde (5.41 ± 0.12) mentioned significantly higher number of TWS as socio-economically important than Otamari (5.44 ± 0.13) people. Natimba (4.93 ± 0.11) mentioned less important threatened woody species (Fig. 8).

The similarity among socio-demographic factors (age, zone, and sociolinguistic group) as regards the composition of TWS cited by respondents was revealed by the matrix of Jaccard’s similarity coefficient (Table 3). Threatened species composition varied significantly among age categories (*R* = 0.057, *p* = 0.0009). Coefficient of similarity between young and old people (0.374) was significantly lower resulting in a high difference between the species mentioned by younger and older informants. Moreover, the composition of TWS mentioned by respondent was very similar between adult and old, and to some extent between young and adult (Jaccard’s coefficients of 0.783 and 0.431, respectively). Analysis of similarity among regions was globally significant (*R* = 0.221, *p* = 0.0009). Threatened woody species mentioned by people from West Atakora (WA) were significantly different from those cited either by people from Central Atakora (CA) and people from East Atakora (EA) (Jaccard’s coefficients of 0.318 and 0.368, respectively, Table 3). About half of the species cited by people from WA were also cited by respondents from CA (Jaccard’s coefficient of 0.576). On the other hand, TWS composition also varied significantly among sociolinguistic groups (ANOSIM; *R* = 0.206, *p* = 0.0009). Analysis of similarity coefficient matrix revealed that TWS cited by Bariba informants were significantly different from those cited by Berba (0.275) and Natimba (0.272); meanwhile, species mentioned by the two latter were relatively more similar from each other (0.418; Table 3). Species cited by Berba were significantly more different than similar to Dendi, Fulfulde, Otamari, and Waama (Jaccard’s similarity coefficients of 0.358, 0.333, 0.355, and 0.306, respectively). Likewise, there was a highly significant difference between Bariba and Otamari (0.319), and Fulfulde and Otamari (0.370). At least 40% of the species cited by Dendi people were similar to those mentioned by Natimba (0.418) and Otamari (0.466) informants. There was no significant difference between Bariba, Dendi, Fulfulde, Otamari, and Waama regarding the species mentioned. Consequently, these sociolinguistic groups knew the same TWS. Overall, there was a great intercultural difference as regards the TWS mentioned by respondents and the greater differentiation was detected between Bariba and Berba, and between Bariba and Natimba.

**Table 3:** Similarity matrix (Jaccard’s coefficients) among sociolinguistic groups as regards the threatened and important woody species

	Bariba	Berba	Dendi	Fulfulde	Natimba	Otamari	Waama
Bariba	–	*0.233* ^***^	*0.317* ^**^	*0.164* ns	*0.224* ns	*0.305* ^***^	*0.283* ^**^
Berba	0.275 ^***^	–	*0.324* ^**^	*0.304* ns	*0.370* ^***^	*0.387* ^***^	*0.483* ^***^
Dendi	0.424 ns	0.358 ^***^	–	*0.345* ^***^	*0.484* ^*^	*0.405* ^**^	*0.444* ns
Fulfulde	0.412 ns	0.333 ^***^	0.558 ns	–	*0.421* ^*^	*0.269* ns	*0.320* ns
Natimba	0.272 ^*^	0.453 ^***^	0.418 ^**^	0.457 ^*^	–	*0.429* ^***^	*0.538* ^**^
Otamari	0.319 ^***^	0.355 ^***^	0.466 ^**^	0.370 ^**^	0.449 ^***^	–	*0.484* ^***^
Waama	0.360 ns	0.306 ^***^	0.491 ns	0.511 ns	0.447 ^**^	0.415 ns	–

Data in italics are Jaccard’s coefficients of important woody species

*ns* non-significant

^*^P value ≤ 0.05, ^**^P value ≤0.01, ^***^P value ≤ 0.001. Differences were tested using Analysis of Similarities (ANOSIM)

Similarity matrix based on Jaccard’s coefficient showed significant differences in the composition of important woody species among age categories (*R* = 0.050; *p* = 0.0020), zones (*R* = 0.109; *p* = 0.0009) and sociolinguistic groups (*R* = 0.130; *p* = 0.0009; Table 2). Species mentioned as important by middle-aged informants were very similar to those cited by older people (Jaccard’s coefficient of 0.703). Therefore, adults knew as much important species than old people while young informants knew lesser important woody species compared to adults and older informants (Jaccard’s coefficients of 0.403 and 0.328, respectively). The coefficient of similarity between East and West Atakora was significantly lower (0.299) likewise between EA and CA (0.362). The coefficient of similarity between Central and the West Atakora was the highest (0.452). Thus, people from EA knew very different important species compared to people from WA, and the latter knew more similar species than informants from CA. The analysis of similarity (Table 3) revealed that species cited by Bariba people as important were highly different from those cited by Berba and by Waama informants. Species composition as mentioned by respondents was moderately similar among Bariba, Berba, Dendi, Fulfulde, Natimba, and Otamari (Jaccard’s coefficient between 0.305 and 0.429). Almost half of the species mentioned by Waama people were similar to those mentioned by Berba, Natimba, Otamari, and Dendi. Therefore, there was high to moderate differences in the important woody species composition with respect to sociolinguistic groups and the higher differences were found between Bariba and Berba, and between Bariba and Waama.

### Potential substitutes of threatened woody species: between-region differences

**Fig. 9:**
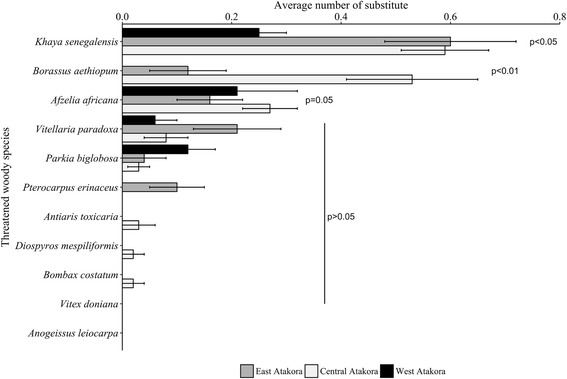
Potential substitutes for the common more threatened woody species across regions. *p* = *p* value from the generalized linear model (GLM) of Poisson/quasi-Poisson

Differences in substitute species were assessed for the most threatened woody species common to the three regions (Table 4). Overall, average number of substitute species significantly differed among regions for *K. senegalensis*, *B. aethiopum*, and *A. africana* (GLM; *p* ≤ 0.05; Fig. 9). In East Atakora (EA), the average number of substitute species was highest for *K. senegalensis* (0.6 ± 0.12), followed by *V. paradoxa* (0.21 ± 0.08), *A. africana* (0.16 ± 0.06), and *B. aethiopum* (0.12 ± 0.07), while in Central Atakora (CA), *K. senegalensis* (0.59 ± 0.08), *B. aethiopum* (0.53 ± 0.12), and *A. africana* (0.27 ± 0.05) respectively had the higher number of substitute. In West Atakora (WA), *K. senegalensis* (0.25 ± 0.05) had the greater average number of substitute, followed by *A. africana* (0.21 ± 0.11) while no substitute was mentioned for *B. aethiopum*. Therefore, informants from EA and those from CA knew more substitutes of *K. senegalensis* than those from WA. Moreover, people from CA knew in average more substitute of *B. aethiopum* than people from the other regions. Although the average number of substitutes of *A. africana* cited by informants were relatively similar among regions, people from CA mentioned more substitute species than those from WA and EA respectively. Average number of substitute species did not vary for *V. paradoxa*, *P. biglobosa*, *P. erinaceus*, *A. toxicaria*, *D. mespiliformis*, and *B. costatum* (GLM; *p* > 0.05; Fig. 9). No substitute species was cited for *V. doniana* and *A. leiocarpa* in the three regions.

**Table 4:** Most threatened woody species common to the three zones

Threatened woody species	Zones		
	EA	CA	WA
*Afzelia africana* Pers.	x	x	x
*Anogeissus leiocarpa* (DC.) Gill. & Perr.	x	x	x
*Antiaris toxicaria* (Engl.) C. C. Berg	x	x	x
*Bombax costatum* Pellegrin & Vuillet	x	x	x
*Borassus aethiopum* Mart.	x	x	x
*Diospyros mespiliformis* Hochst. Ex A.DC.	x	x	x
*Khaya senegalensis* (Desv.) A. Juss	x	x	x
*Parkia biglobosa* (Jacq.)G.Don	x	x	x
*Pterocarpus erinaceus* Poir.	x	x	x
*Vitellaria paradoxa* C.F.Gaertn	x	x	x
*Vitex doniana* Sweet	x	x	x
*Ceiba pentandra* (L) Geartn	x	x	
*Elaeis guineensis* Jacq.	x	x	
*Milicia excelsa* (Welw.) C.C. Berg	x	x	
*Adansonia digitata* L.	x		x
*Tamarindus indica* L.	x		x
*Isoberlinia doka* Craib & Stapf	x		
*Isoberlinia tomentosa* (Harms) Craib & Stapf	x		
*Kigelia africana* (Sprague) Bidgood & Verdc	x		
*Raphia sudanica* A. Chev.	x		
*Afraegle paniculata* (Schum.)		x	x
*Prosopis africana* (Guill. & Perr.)Taub.		x	x
*Bombax buonopozense* Beauv.		x	
*Ficus platyphylla* Del.		x	
*Oxytenanthera abyssinica* (A.Rich.)		x	
*Zanthoxylum zanthoxyloides* (Lam.) B.Zepernick & F.K. Timler	x		
*Detarium microcarpum* Guill. & Perr.			x
*Ficus gnaphalocarpa* Steud. Ex Miq.			x
*Lannea microcarpa* Engl & K. Krause			x
*Mitragyna inermis* (Willd.) Kuntze			x
*Sclerocarya birrea* (Sond.) Kokwaro			x

*EA* East Atakora, *CA* Central Atakora, *WA* West Atakora

**Fig. 10:**
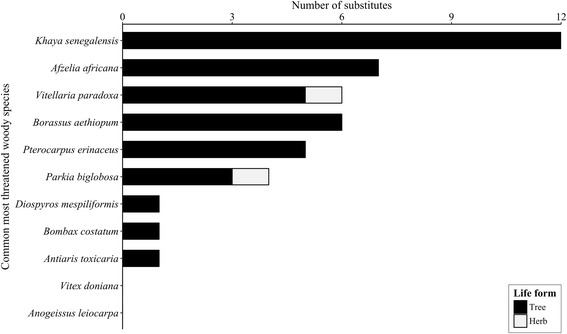
Number and life form of the potential substitutes for each common more threatened woody species

Most of substitutes were also woody species except for *Glycine max* and *Arachis hypogaea*, two herbs that were substitute for *P. biglobosa* and *V. paradoxa* respectively (Fig. 10, Table 5). Substitute species more cited by respondents varied across regions. *P. erinaceus* was mainly mentioned as substitute of *A. africana* in EA (25.29% of respondents) and to some extent in the CA (5.77%) while *T. indica* was mostly cited in WA (3.95% of informants, Table 5). *Khaya* spp. and *P. erinaceus* were equally more cited as substitute of *B. aethiopum* in CA (cited by 14.94% of informants). More cited substitute species for *K. senegalensis* were *I. doka* (19.24%) and *I. tomentosa* (13.46%) in EA, *P. erinaceus* and *A. africana* in CA (40.23 and 14.94%, respectively), and *P. erinaceus* in WA (23.68%). The most cited substitute species for *P. biglobosa* was *A. digitata* in the Atakora chain (2.30%), while *A. digitata* and *G. max* were respectively more cited in WA (5.26 and 2.63%). For *V. paradoxa*, people mentioned more *P. butyracea* as substitute in EA (6.73%) and in CA (4.60%) while *A. hypogaea* was most cited in WA (2.63%; Table 5). Overall, *P. erinaceus* was the most cited substitute species, mentioned by 38.2% of informants. The species was mainly mentioned as substitute for *K. senegalensis*, *A. africana*, and *B. aethiopum* (22.47, 10.49, and 5.24% of respondents, respectively). The second more cited substitute species was *Isoberlinia doka* (7.49% of all informants), followed by *A. africana* (6.74%), both mentioned for *K. senegalensis.*

**Table 5:** Frequency of substitute mentioned by respondents for each more threatened woody species

Common more threatened species	Substitutes	LF	Zones (%)			
			EA (*n* = 104)	CA (*n* = 87)	WA (*n* = 76)	Whole (%)
*Afzelia africana*	*Khaya* spp	Tree	1.92	0.00	0.00	0.75
	*Tectona grandis* L.f.	Tree	0.96	0.00	0.00	0.37
	*Eucalyptus* spp	Tree	0.96	0.00	0.00	0.37
	*Leucaena leucocephala* (Lam.)de Wit	Tree	1.92	0.00	0.00	0.75
	*Pterocapus erinaceus* Poir.	Tree	5.77	25.29	0.00	10.49
	*Isobelinia* spp	Tree	0.96	0.00	0.00	0.37
	*Tamarindus indica* L.	Tree	0.00	0.00	3.95	1.12
*Anogeissus leiocarpa*	*–*		0.00	0.00	0.00	0.00
*Antiaris toxicaria*	*Pterocapus erinaceus* Poir.	Tree	0.00	1.15	0.00	0.37
*Bombax costatum*	*Daniellia oliveri* (Rolfe)Hutch. & Dalziel	Tree	0.00	1.15	0.00	0.37
*Borassus aethiopum*	*Elaeis guineensis* Jacq.	Tree	0.96	0.00	0.00	0.37
	*Anogeissus leiocarpa* (DC.) Gill. & Perr.	Tree	0.96	0.00	0.00	0.37
	*Khaya* spp	Tree	0.96	14.94	0.00	5.24
	*Afzelia africana* Pers.	Tree	0.96	1.15	0.00	0.75
	*Pterocapus erinaceus* Poir.	Tree	0.96	14.94	0.00	5.24
	*Isobelinia* spp	Tree	0.96	0.00	0.00	0.37
*Diospyros mespiliformis*	*Pterocarpus erinaceus* Poir.	Tree	0.00	1.15	0.00	0.37
*Khaya senegalensis*	*Acacia sieberiana* DC.	Tree	0.96	0.00	0.00	0.37
	*Afzelia africana* Pers.	Tree	4.81	14.94	0.00	6.74
	*Pterocarpus erinaceus* Poir.	Tree	6.73	40.23	23.68	22.47
	*Khaya* spp	Tree	0.00	0.00	0.00	0.00
	*Borassus aethiopum* Mart.	Tree	0.00	2.30	0.00	0.75
	*Ekebergia capensis* Sparrm.	Tree	19.23	0.00	0.00	0.75
	*Isoberlinia doka* Craib & Stapf	Tree	13.46	0.00	0.00	7.49
	*Isoberlinia tomentosa* (Harms) Craib & Stapf	Tree	4.81	0.00	0.00	5.24
	*Tectona grandis* L.f.	Tree	5.77	0.00	0.00	2.25
	*Leucaena leucocephala* (Lam.)de Wit	Tree	1.92	0.00	0.00	0.75
	*Pseudocedrela kotschyi* (Schweinf.) Harms	Tree	6.73	0.00	0.00	2.62
*Parkia biglobosa*	*Adansonia digitata* L.	Tree	0.00	2.30	5.26	2.25
	*Glycine max* (L.)Merr.	Herb	0.00	0.00	2.63	0.75
	*Prosopis africana* (Guill. & Perr.)Taub.	Tree	0.96	0.00	0.00	0.37
	*Acacia auriculiformis* A.Cunn. ex Benth.	Tree	0.96	0.00	0.00	0.37
*Pterocarpus erinaceus*	*Acacia sieberiana* DC.	Tree	0.96	0.00	0.00	0.37
	*Isoberlinia* spp.	Tree	1.92	0.00	0.00	0.75
	*Tectona grandis* L.f.	Tree	0.96	0.00	0.00	0.37
	*Khaya* spp	Tree	0.96	0.00	0.00	0.37
	*Leucaena leucocephala* (Lam.)de Wit	Tree	1.92	0.00	0.00	0.75
*Vitellaria paradoxa*	*Anacardium occidentale* L.	Tree	0.96	0.00	0.00	0.37
	*Mangifera indica* L.	Tree	0.96	0.00	0.00	0.37
	*Arachis hypogaea* L.	Herb	0.00	0.00	2.63	0.75
	*Pentadesma butyracea* Sabine	Tree	6.73	4.60	0.00	4.12
	*Acacia sieberiana* DC.	Tree	0.96	0.00	0.00	0.37
	*Prosopis africana* (Guill. & Perr.)Taub.	Tree	0.96	0.00	0.00	0.37
*Vitex doniana*	*–*		0.00	0.00	0.00	0.00

*LF* life form, *EA* East Atakora, *CA* Central Atakora, *WA* West Atakora

**Fig. 11:**
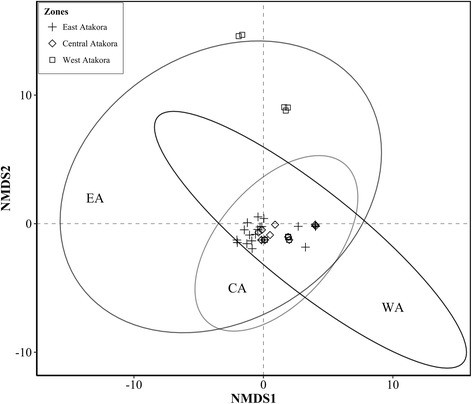
Ordination diagram of a NMDS of substitutes of 11 threatened woody species in three zones. The stress value was 0.002, and confidence ellipses were built at 95% confidence level

There was a weak discrimination of substitute species across regions (Fig. 11). A full overlap of confidence ellipses was observed between EA and CA indicating a high similarity between substitute species mentioned in these two regions. In contrast, overlapping of confidence ellipse was partial between WA and EA or CA indicating that substitute species composition was relatively distinct between WA and CA or between WA and EA.

## Discussion

This study assessed the traditional knowledge on threatened woody species (TWS) in the Atakora mountain chain region of Benin and its relationship with socio-demographic attributes of locals. It further evidences the substitute species as resource depletion adaptation.

The diversity of TWS in the Atakora chain region is estimated at 117 species, representing about 4.17% of the national flora of Benin estimated at 2807 species [43]. About 12% of the identified TWS are red listed in Benin and in IUCN list, with *Afraegle paniculata*, *Afzelia africana*, *Khaya grandifoliola*, *K. senegalensis*, *Milicia excelsa*, *Pterocarpus erinaceus*, and *Triplochiton scleroxylon*, highly endangered in the country, the others being vulnerable [16]. These observations are supporting the status of Atakora region and its mountain chain, known to be a hotspot of biodiversity in Benin [15], hosting three endemic genera (*Vitellaria*, *Pseudocedrela*, *and Haematostaphis*) to the Sudanian zone, the two Beninese’s endemic plant species (*Cyperus beninensis* and *Ipomoea beninensis*), as well as *Thunbergia atacorensis*, an endangered species endemic to the inselbergs of Benin and Togo [16, 17].

The identified TWS are of different socio-economic importance to local people in Atakora region. *K. senegalensis*, *P. biglobosa*, *V. paradoxa*, and *V. doniana* were reported to be of high socio-economic importance to local people due to their use for multiple purposes including food, medicine, and culture, congruently to recent observation of Heubach [44]. Indeed, in the Atakora region, *K. senegalensis* is abundantly used as timber, fodder, and service wood and to some extent as medicine [45]. *P. biglobosa* is reported to contribute to up to 53% of income of nearly all households in the region of Atakora chain. Its fermented seeds are even richer in protein than meat [46] and are highly sought for seasoning soup [1]. *V. doniana* is a popular leafy vegetable with high economic importance, which sweet prune-like fruits are largely consumed and even sold whereas other parts of the plant are used in the treatment of various ailments [47]. *V. paradoxa* fruit’s pulp is edible and widely consumed by local people. The shea-butter obtained by processing its kernels is used in traditional medicine and cosmetic industry and is at the core of important national and international economic activities while its tree serves as fuelwood and building material [48]. However, the traditional ecological knowledge of TWS and their related socio-economic importance were influenced by geographical location, generation, and sociolinguistic group, supporting the general assumption that the relative importance of species and forest products to populations is context dependent [49]. In this study, there was a relatively higher traditional knowledge on TWS in East Atakora in comparison to other parts of the Atakora region. This discrepancy may be related to the availability of plant resources [30] and suggests that woody species might be more diverse and abundant in the East region than the others. Similarly, *K. senegalensis* and *P. biglobosa* were found to be most important TWS in the East Atakora while *V. paradoxa* and *V. doniana* were reported to be the most important in Central and West Atakora. The discrepancy in traditional ecological knowledge and its related importance were also observed within regions, ruled by age and sociolinguistic groups. With regard to age categories, the traditional knowledge on TWS was found to be higher with older people, evidencing a life learning process [50]. Finally, as also observed by Fandohan et al. [51] for *Tamarindus indica* in the same region, the traditional knowledge related to TWS varied among sociolinguistic groups, evidencing thus cultural-specific knowledge on TWS. As a result, future strategies for the conservation of TWS should account for geographical location, age, gender, and sociolinguistic groups to copy with the differences.

Although local people in Atakora region showed extended knowledge on TWS, paradoxically, not all the TWS are of socio-economic importance to local people. These observations suggest that the threats to some woody species in Atakora regions may not be from direct pressure (overexploitation) from local people, but rather likely from indirect anthropogenic actions (e.g., forest degradation, urbanization), from global change (climate change, large conversion of landscape into farmlands), or from external sources (users from other regions, riparian to Atakora regions). Therefore, future strategies should take into account these diverse and specific threats to TWS.

Whatever the threat sources, the TWS are under pressure with declining populations. Local people in Atakora develops TWS depletion adaptation strategy by using substitute plant species. The number of potential substitutes to TWS was particularly higher for some species (e.g., *K. senegalensis*, *A. africana*, and *B. aethiopum*), indicating a relatively high level of uses of these resources in this region and their ongoing rarefaction due to high human pressure. The substitutes to a given TWS varied with regions. For instance, *P. erinaceus* and *T. indica* were substitutes to *A. africana* in EA and WA, respectively, suggesting then that the mechanism of TWS substitution is spatial, probably driven socio-cultural considerations, availability and abundance of the substitute, and capacity of the substitute to adequately compensate and maximize the utility devoted to the primary TWS. In addition, the mechanism of TWS substitution appears to be temporally dynamic. Indeed, *P. erinaceus* reported to be substituted to *A. africana* and *K. senegalensis* during this study is getting very rare in the Atakora region with high conservation issues [52] and being replaced by *Isoberlinia doka* and *I. tomentosa* (Fabaceae) also mentioned as substitutes*.*

From this study, the substitute species were selected mostly among the same pool of life form (tree and woody species), genera, or families to maximize the utility of the substitute. However, while guarantying the satisfaction, plant selection from the same pool may reduce the freedom level of choice and contribute to the selective depletion of plant groups (genera or families). To be sustainable, the mechanism of TWS substitution may go beyond the same pool and explore other functional groups. For instance, in Atakora regions, *P. biglobosa* was substituted with the soybean *Glycine max* while *V. paradoxa* was replaced by *Arachis hypogea*. The substitution pattern of *P. biglobosa* makes sense as soybean is rich enough to compensate the protein supply of the fermented and processed seeds of *P. biglobosa* which is a popular ingredient locally used in sauce.

Overall, the substitution mechanism is not always a sustainable panacea for controlling the depletion of TWS, especially by selecting in a same pool of threatened species. However, the substitution of a perennial woody species by an annual plant could represent a sustainable alternative to slow down the decline of the TWS.

## Conclusion

The study provides data on the diversity of, and local ecological knowledge on, threatened woody species currently found in the Atakora mountain chain region in Benin. Their families and genera vary with respect to the zone and informants showed a good level of knowledge about these species. Therefore, community-based management programs involving people from different areas, cultures, and ages for gender-sensitive experience sharing will be a judicious strategy for sustainable conservation of those threatened woody resources and their ecosystem in the study area. The most threatened species including *Khaya senegalensis*, *Pterocarpus erinaceus*, *Borassus aethiopum*, *Anogeissus leiocarpa*, and *Diospyros mespiliformis* need urgent conservation actions. We recommend ex-situ conservation of these species while promoting their integration into agroforestry-based systems.

Local communities rely on a variety of substitutes as adaptation measure to the rarefaction of daily used species. The choice of surrogate is dynamic and evolves in space and time. Therefore, a threatened and socio-economically important species in one region may be a potential substitute in another, and minor species of today will likely become of great importance in the future. However, people develop unsustainable practices that compromise the survival of minor species which are prone to extinction, and in doing so, they may run out of substitutes later. Strategies for conservation of woody species should then target not only the socio-economically important threatened species but also the minor species, for the next generations. Furthermore, the central government, scientists, NGOs, and actors at different levels must be aware of their responsibility and crucial role in educating people to conserve nature as our universal common inheritance.

## Abbreviations

ANOSIM: Analysis of similarities
ANOVA: Analysis of variance
CA: Central Atakora
EA: East Atakora
FAO: Food and Agriculture Organization
GLM: Generalized linear model
IUCN: International Union for Conservation of Nature
NMDS: Non-metric multidimensional scaling
SNK: Student Newman Keuls
TEK: Traditional ecological knowledge
TWS: Threatened woody species
WA: West Atakora

## References

[1] Vodouhê FG, Adégbidi A, Coulibaly O, Sinsin B (2011). Parkia biglobosa (Jacq.) R. Br. Ex Benth. harvesting as a tool for conservation and source of income for local people in Pendjari Biosphere Reserve. Acta Botanica Gallica.

[2] Sinsin B, Matig OE, Assogbadjo A, Gaoué O, Sinadouwirou T (2004). Dendrometric characteristics as indicators of pressure of Afzelia africana Sm. dynamic changes in trees found in different climatic zones of Benin. Biodivers Conserv.

[3] Shackleton S, Delang CO, Angelsen A. From subsistence to safety nets and cash income: exploring the diverse values of non-timber forest products for livelihoods and poverty alleviation. In: Non-timber forest products in the global context. Berlin: Springer; 2011. p. 55–81.

[4] Shackleton S, Gumbo D, Chidumayo EN, Gumbo DJ (2010). Contribution of non-wood forest products to livelihoods and poverty alleviation. The dry forests and woodlands of Africa: managing for products and services.

[5] Gockowski J, Sonwa D (2011). Cocoa intensification scenarios and their predicted impact on CO2 emissions, biodiversity conservation, and rural livelihoods in the Guinea rain forest of West Africa. Environ Manag.

[6] Gibbs HK, Ruesch AS, Achard F, Clayton MK, Holmgren P, Ramankutty N, Foley JA (2010). Tropical forests were the primary sources of new agricultural land in the 1980s and 1990s. Proc Natl Acad Sci.

[7] Amagnide AG, Salako V, Hounsode MD, Sinsin F, Orékan V, Dan C, Kakaï RG (2015). Ecological consequences of anthropogenic pressure in Wari-Maro Forest Reserve (Benin, West Africa). J Agric Environ Int Dev (JAEID).

[8] Orekan VO. Implementation of the local land-use and land-cover change model CLUE-s for Central Benin by using socio-economic and remote sensing data. Bonn: Ph.D. Thesis. Shaker Verlag; 2008.

[9] Leh MD, Matlock MD, Cummings EC, Nalley LL (2013). Quantifying and mapping multiple ecosystem services change in West Africa. Agric Ecosyst Environ.

[10] Poorter L. Biodiversity of West African forests: an ecological atlas of woody plant species. Wallingord: CABI Publishing; 2004.

[11] FAO (2011). Situation des forêts du monde.

[12] Orekan V, Tente B, Houndagba CJ, Abdoulaye D. Landcover and vegetation cover dynamics. In: Sinsin B, Kampmann D, editors. Biodiversity Atlas of West Africa, volume 1, vol. I. Franckfurt University: Cotonou & Franckfurt/Main; 2010.

[13] CILSS (2016). Landscapes of West Africa––a window on a changing world.

[14] Glèlè Kakaï R, Assogbadjo A, Sinsin B, Pelz D (2009). Structure spatiale et régénération naturelle de Pterocarpus erinaceus Poir en zone soudanienne au Bénin. Rev Ivoirienne Sci Technol.

[15] Wala K (2010). La végétation de la chaîne de l'Atakora au Bénin: diversité floristique, phytosociologie et impact humain. Acta Botanica Gallica.

[16] Neuenschwander P, Sinsin B, Goergen G. Protection de la Nature en Afrique de l'Ouest: Une Liste Rouge pour le Bénin. Nature Conservation in West Africa: red list for Benin. International Institute of Tropical Agriculture. Ibadan: International Institute of Tropical Agriculture; 2011.

[17] Dourma M, Batawila K, Guelly KA, Bellefontaine R, Foucault B, Akpagana K (2012). La flore des forêts claires à Isoberlinia spp. en zone soudanienne au Togo Titre courant: Flore des forêts claires à Isoberlinia. Acta Botanica Gallica.

[18] Paré S, Savadogo P, Tigabu M, Ouadba JM, Odén PC (2010). Consumptive values and local perception of dry forest decline in Burkina Faso, West Africa. Environ Dev Sustain.

[19] Sop T, Oldeland J (2013). Local perceptions of woody vegetation dynamics in the context of a ‘greening Sahel’: a case study from Burkina Faso. Land Degrad Dev.

[20] Abdala T, Eshetu G, Worku A (2017). Participatory assessment of threatened forest species in Hararge Area, Eastern Ethiopia: community based participatory.

[21] Tabuti JR, Mugula BB (2007). The ethnobotany and ecological status of Albizia coriaria Welw. ex Oliv. in Budondo Sub-county, eastern Uganda. Afr J Ecol.

[22] Ayyanar M, Ignacimuthu S (2011). Ethnobotanical survey of medicinal plants commonly used by Kani tribals in Tirunelveli hills of Western Ghats, India. J Ethnopharmacol.

[23] Paré S (2008). Land use dynamics, tree diversity and local perception of dry forest decline in Southern Burkina Faso, West Africa.

[24] Ouinsavi C, Sokpon N, Bada O (2005). Utilization and traditional strategies of in situ conservation of iroko (Milicia excelsa Welw. CC Berg) in Benin. For Ecol Manag.

[25] Andrade G, Rhodes J. Protected areas and local communities: an inevitable partnership toward successful conservation strategies? Ecol Soc. 2012;17(4):14. 10.5751/ES-05216-170414.

[26] Kiptot E, Hebinck P, Franzel S, Richards P (2007). Adopters, testers or pseudo-adopters? Dynamics of the use of improved tree fallows by farmers in western Kenya. Agric Syst.

[27] Adomou AC. Vegetation patterns and environmental gradient in Benin: implications for biogeography and conservation. Wageningen: Ph.D. Thesis. Wageningen University; 2005.

[28] Adam K, Boko M (1993). Le Bénin.

[29] The IUCN Red List Categories and Criteria (version 3.1). http://www.iucnredlist.org/static/categories_criteria_3_1. Accessed 14 Feb 2018.

[30] de Albuquerque UP (2006). Re-examining hypotheses concerning the use and knowledge of medicinal plants: a study in the Caatinga vegetation of NE Brazil. J Ethnobiol Ethnomed.

[31] United Nations, Departement of Economic and Social Affairs, Population Division (2013). World population ageing 2013. ST/ESA/SER.A/348.

[32] Dytham C. Choosing and using statistics: a biologist’s guide. 3rd ed. York: Wiley; 2011.

[33] Zar JH. Biostatistical analysis: Pearson New International Edition. Pearson Higher Ed: Illinois; 2013.

[34] Ogle D (2017). FSA: fisheries stock analysis. R Package Version.

[35] R-Core-Team (2017). R: a language and environment for statistical computing.

[36] Chang H-J, Huang K-C, Wu C-H (2006). Determination of sample size in using central limit theorem for weibull distribution. Inf Manag Sci.

[37] Lê S, Josse J, Husson F (2008). FactoMineR: an R package for multivariate analysis. J Stat Softw.

[38] Quinlan M (2005). Considerations for collecting freelists in the field: examples from ethobotany. Field Methods.

[39] Albuquerque UP, Ramos MA, de Lucena RFP, Alencar NL. Methods and techniques used to collect ethnobiological data. New York: Springer, Humana Press; 2014.

[40] Clarke KR (1993). Non-parametric multivariate analyses of changes in community structure. Austral Ecol.

[41] Oksanen J, Blanchet FG, Friendly M, Kindt R, Legendre P, McGlinn D, Minchin PR, O’Hara B, Simpson GL, Solymos P (2017). Vegan: community ecology package. R package version 24-3.

[42] Bray JR, Curtis JT (1957). An ordination of the upland forest communities in southern Wisconsin. Ecol Monogr.

[43] Akoègninou A, van der Burg WJ, van der Maesen LJG (2006). Flore Analytique du Bénin.

[44] Heubach K, Schumann K, Hahn K (2016). Substitutes for seeds of Vitellaria paradoxa, Parkia biglobosa and Adansonia digitata used for nutrition by five major ethnic groups in Benin, West Africa. Flora et Vegetatio Sudano-Sambesica.

[45] Houehanou TD, Assogbadjo AE, Glèlè Kakaï R, Houinato M, Sinsin B (2011). Valuation of local preferred uses and traditional ecological knowledge in relation to three multipurpose tree species in Benin (West Africa). Forest Policy Econ.

[46] Kronborg M, Ilboudo J-B, Bassolé IHN, Barfod AS, Ravn HW, Lykke AM (2014). Correlates of product quality of soumbala, a West African non-timber forest product. Ethnobot Res Appl.

[47] Dadjo C, Assogbadjo AE, Fandohan B, Kakaï RG, Chakeredza S, Houehanou TD, Van Damme P, Sinsin B (2012). Uses and management of black plum (Vitex doniana sweet) in Southern Benin. Fruits.

[48] Sinsin B, Kampmann D, Thiombiano A, Konaté S (2010). Atlas de la Biodiversité de l’Afrique de l’Ouest.

[49] Timko J, Waeber P, Kozak R (2010). The socio-economic contribution of non-timber forest products to rural livelihoods in Sub-Saharan Africa: knowledge gaps and new directions. Int For Rev.

[50] de Albuquerque UP, Soldati GT, Sieber SS, Ramos MA, de Sá JC, de Souza LC (2011). The use of plants in the medical system of the Fulni-ô people (NE Brazil): a perspective on age and gender. J Ethnopharmacol.

[51] Fandohan B, Assogbadjo AE, Glèlè Kakaï R, Kyndt T, De Caluwé E, Codjia JTC, Sinsin B (2010). Women’s traditional knowledge, use value, and the contribution of tamarind (*Tamarindus indica* L.) to rural households’ cash income in Benin. Econ Bot.

[52] Akpona JDT, Assogbadjo AE, Fandouan AB, Kakai RG. Inventory and multicriteria approach to identify priority commercial timber species for conservation in Benin. Bois et forêts des tropiques. 2017;333(3):5–16.

